# Novel systems to study vector-pathogen interactions in malaria

**DOI:** 10.3389/fcimb.2023.1146030

**Published:** 2023-05-26

**Authors:** Marina Parres-Mercader, Alena Pance, Elena Gómez-Díaz

**Affiliations:** ^1^ Instituto de Parasitología y Biomedicina López-Neyra, Consejo Superior de Investigaciones Científicas (IPBLN, CSIC), Granada, Spain; ^2^ School of Life and Medical Sciences, University of Hertfordshire, Hatfield, United Kingdom

**Keywords:** mosquito, Plasmodium, organoids, tissue explant, membrane feeding assay (MFA), *Anopheles*

## Abstract

Some parasitic diseases, such as malaria, require two hosts to complete their lifecycle: a human and an insect vector. Although most malaria research has focused on parasite development in the human host, the life cycle within the vector is critical for the propagation of the disease. The mosquito stage of the *Plasmodium* lifecycle represents a major demographic bottleneck, crucial for transmission blocking strategies. Furthermore, it is in the vector, where sexual recombination occurs generating “*de novo*” genetic diversity, which can favor the spread of drug resistance and hinder effective vaccine development. However, understanding of vector-parasite interactions is hampered by the lack of experimental systems that mimic the natural environment while allowing to control and standardize the complexity of the interactions. The breakthrough in stem cell technologies has provided new insights into human-pathogen interactions, but these advances have not been translated into insect models. Here, we review *in vivo* and *in vitro* systems that have been used so far to study malaria in the mosquito. We also highlight the relevance of single-cell technologies to progress understanding of these interactions with higher resolution and depth. Finally, we emphasize the necessity to develop robust and accessible *ex vivo* systems (tissues and organs) to enable investigation of the molecular mechanisms of parasite-vector interactions providing new targets for malaria control.

## 1 Introduction

Mosquitoes are responsible for the transmission of many life-threatening diseases, causing millions of deaths every year. Malaria is one of the deadliest infectious diseases affecting half of the world’s population and causing over half a million deaths per year (WHO, 2022). It is caused by apicomplexan parasites of the *Plasmodium* genus and is transmitted by the bite of a female *Anopheles* mosquito. The parasite survival in the mosquito is necessary for the spread of malaria and is therefore a target for the development of transmission blocking strategies (Yu et al., 2022).

**Figure 1:**
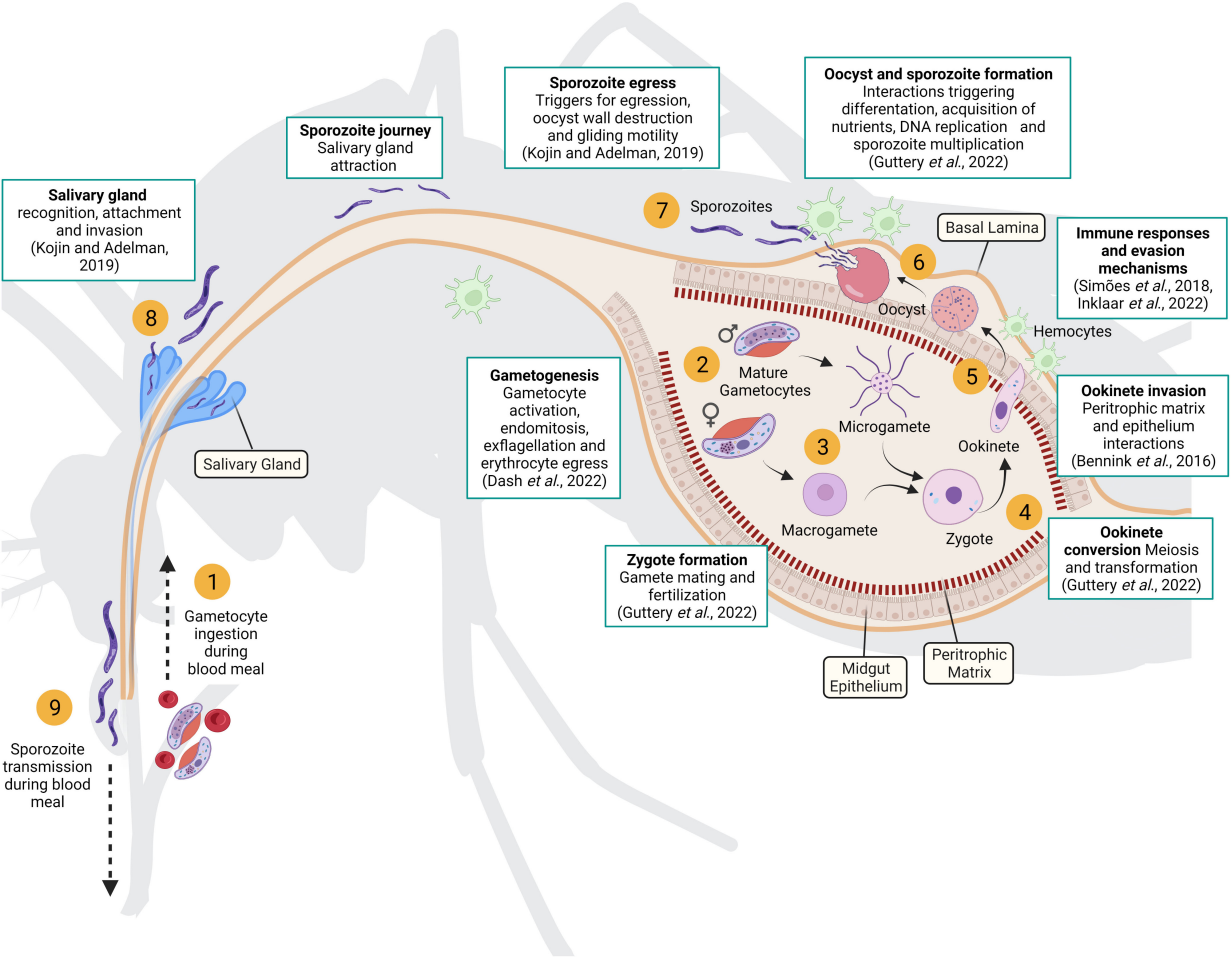
Parasite life cycle in the mosquito vector. Gametocytes ingested during a blood meal (1) are activated in the mosquito midgut and differentiated into female and male gametes (2). Fertilization occurs when two gametes fuse (3) resulting in a diploid zygote that initiates meiosis until it differentiates into an ookinete (4). Mature ookinetes can penetrate the peritrophic matrix and midgut epithelium to reach the basal lamina (5), where they develop into oocysts (6). Inside the oocyst, hundreds of sporozoites are produced and then released into the mosquito hemocoel (7). Sporozoites migrate to the salivary glands (8) and are injected by the mosquito into a new host (9). The white boxes highlight key processes underpinning parasite development in the mosquito that require further investigation (recent reviews of each key processes are included). Created with Biorender.com.

The life cycle of the parasite in the vector begins when a mosquito bites an infected human host and ingests the sexual parasite stages, or gametocytes, along with the blood meal (**Figure 1**). Gametocytes exposed to the midgut environment are activated, egress from the erythrocytes and differentiate into gametes (Dash et al., 2022). Each male gametocyte divides and generates eight flagellated microgametes in a process called exflagellation. Meanwhile female gametocytes mature and form a single rounded immotile macrogamete. Fertilizations occurs when two gametes fuse resulting in a diploid zygote that initiates meiosis until differentiate into an ookinete (Guttery et al., 2022). This transformation involves several morphological changes that confer the mature ookinete the ability to glide and cross two physical barriers: the peritrophic matrix, secreted by midgut cells after ingestion of a blood meal, and the midgut epithelium. At this point, peritrophic matrix disruption and midgut cell damage triggers the mosquito’s immune responses, that results in an important reduction of the parasite population(Simões et al., 2018). Selective forces are very strong, and to survive to this major bottleneck, malaria parasites have developed immune evasion strategies (Inklaar et al., 2022). When the ookinete reaches the basal side of the midgut, it undergoes another morphological change rounding up to form an oocyst. Inside the oocyst, hundreds of sporozoites are produced by mitosis and then released into the mosquito hemocoel. The sporozoites invade the salivary glands(Kojin and Adelman, 2019), waiting to be injected into a new host by the next mosquito bite.

Despite important advances in the identification of the molecular interactions between the parasite and the mosquito (Bennink et al., 2016), we are still far from a complete understanding of the factors and mechanisms that are critical for the development and the survival of the parasite during the sporogonic cycle: gamete mating, zygote formation and recombination, ookinete invasion of the mosquito midgut and immune protection, oocyst multiplication and growth and sporozoite differentiation and activation in the salivary glands (Guttery et al., 2022). Furthermore, the mosquito environment is highly heterogenous from one infection to another, in terms of immune responses, physiology and behavior; depending on the vector species, the mosquito genotype, the type and number of blood meals and the external environment. This variation imposes strong selective constraints favoring phenotypic variation and rapid adaptation in the parasite population (Ruiz and Gómez-Díaz, 2019). How this variation is generated and what are the underlying mechanisms is still unknown.

Contrary to the malaria blood cycle that has been largely studied *in vitro*, the study of the cycle in the vector has been traditionally limited to the experimental infection of the mosquitoes in the laboratory (Blagborough et al., 2013). Although this *in vivo* system recapitulates the natural interactions, it is very heterogenous and complex to scrutinize the regulatory mechanisms and understand the fine detail of key parasite developmental processes like meiosis. Furthermore, since more than half of *Plasmodium* genes are essential for asexual blood-stage development or transmission, these cannot be targeted using knockout methods (Bushell et al., 2017; Zhang et al., 2018). Functional genetics studies in *Plasmodium* targeting essential genes rely on generating conditional gene knockdowns. Amongst these, ligand-activated systems are commonly used in erythrocytic stages, but pose the challenge of a precise and controlled delivery of the effector molecule into the mosquito compartment, as well as the potential toxic effects in both organisms (Kudyba et al., 2021). Therefore, a robust, flexible and effective conditional knockdown systems for sporogonic stages is still a major hurdle in the field.

Altogether, there are many unknowns about *Plasmodium* development and adaptation in the mosquito, as well as of the responses of the mosquito to an infection. However, no suitable *in vitro* or *ex vivo* models capable of mimicking the complexity and dynamics of a malaria infection have been developed yet. More generally, this lack of suitable insect study models is generalizable to other pathogens and severely limits our ability to fight vector-borne diseases.

In this contribution we review the different systems (*in vivo*, *in vitro*) that are available to study the parasite life-cycle in the mosquito, highlight the strengths, limitations and recent advances. We contend that the development of novel *ex vivo* mosquito systems, has the potential to advance in our knowledge of pathogen-vector interactions providing novel targets to control the propagation of the disease.

## 2 The mosquito *in vivo* model

Experimental mosquito infection is widely used in malaria research as it recapitulates the natural infection, allowing the study of parasite development, mosquito responses to the infection as well as their interaction. Despite the many advantages, the heterogeneity and complexity of the organisms involved impose important limitations in terms of scalability, reproducibility and the potential to manipulate the system, which can result in variable performance of the assays, less robust data and a knowledge gap in many research areas (**Figure 1**). Besides, the infrastructure, material and health and safety considerations for experimental mosquito infections are difficult to establish in many laboratories, and the methodologies are highly time-consuming and laborious.

**Figure 2:**
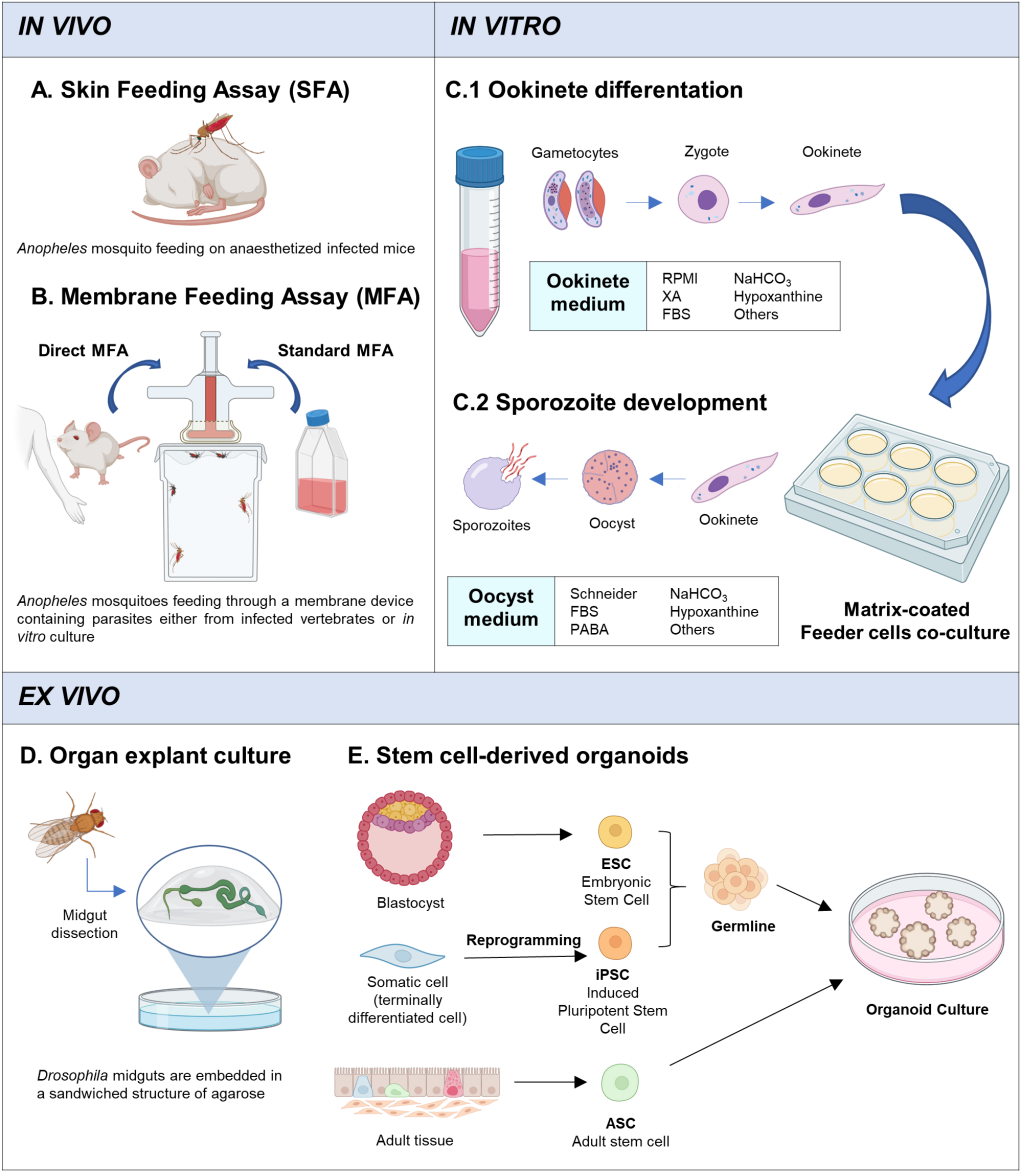
*In vivo*, *in vitro* and *ex vivo* systems. **(A)** Skin Feeding Assay: mosquito bites directly on infected host. **(B)** Membrane Feeding Assay: mosquito feeds through a membrane feeding device which contains infected blood with parasites (gametocytes) from infected-hosts (DMFA) or *in vitro* cultured gametocytes (SMFA). **(C)** Main strategy for *in vitro* culture of Plasmodium mosquito stages. **(C1)** Stage V gametocytes are cultured with ookinete medium for 24h, at 19-26°C (depending on the plasmodium specie). **(C2)** Ookinetes are recovered and pipetted into matrix-coated wells and co-cultured with feeder cells (*Drosophila* S2 cells or others). Oocyst medium is changed periodically until sporozoite development (14-21 days). **(D)** *Ex vivo* culture system developed by Marcchetti et al., (Marchetti et al, 2022). Adult *Drosophila* midguts are dissected and embedded in an agarose sandwich structure with a custom-made culture medium and cultured using an air-media interface. Midguts are maintained alive for up to 3 days. **(E)** Organoids can originate from ESCs, iPSCs, or ASCs. ESCs are derived from the inner cell mass of a blastocyst, iPSCs originate from terminally differentiated cells that have been reprogrammed to become pluripotent stem cell, and, ASCs are isolated from the tissue of interest. ESCs and iPSCs have an additional differentiation step towards the required germline (endoderm, mesoderm, ectoderm). The stem cells are cultured in a defined medium with an extracellular matrix that promotes cell differentiation and 3D structure formation, simulating organ development. Created with Biorender.com.

There are different approaches to infect mosquitoes with disease agents (**Table 1**), by directly biting an infected host, known as skin feeding assay (SFA), or artificially through a membrane feeding device which contains an infective blood meal, called membrane feeding assay (MFA) (**Figures 2A, B**). Although the SFA recapitulates better a natural mosquito infection, it has some limitations compared with MFA (Bousema et al., 2013). Apart from ethical restrictions (especially regarding human patients), the number of mosquitoes that can be fed on each infected vertebrate is limited, as is the rearing capacity of animal facilities. More importantly, there is no control of the gametocyte density in the blood meal or the presence of host serum factors that may interfere with the infection process. A better control of mosquito infection is achieved with MFA, which also allows the use either parasites from infected-hosts (direct membrane feeding assays, DMFA) or *in vitro* cultured parasites (standard membrane feeding assay, SMFA). DMFA better represents the diversity of field parasites but, the heterogeneity of the parasite population can introduce substantial experimental variability. In addition, the accessibility to field samples as well as their handling and transport, can hamper the quality of the assays. Conditions in SMFA are more controlled but gametocyte production capacity could be compromised over time when parasite strains are maintained in continuous *in vitro* culture (Ponnudurai et al., 1982; Brown and Guler, 2020). Moreover, *Plasmodium* species that cannot be cultured *in vitro*, such as *Plasmodium vivax*, *Plasmodium malariae* and *Plasmodium ovale*, are not suitable for this type of assay. These species present culture specificities which we are not able to reproduce yet, such as the particularity of the *P. vivax* to infect reticulocytes instead of mature red blood cells (Thomson-Luque and Bautista, 2021). The mosquito *in vivo* system is the only way to study these species, and DMFA is the strategy used (Miura et al., 2020). Nevertheless, the SMFA is considered the gold standard for evaluating transmission-reducing factors and together with the direct skin feeding assays in mice, is widely and routinely used to study the parasite cycle in the mosquito (Blagborough et al., 2013).

**Table 1:** Systems and strategies used to study mosquito-*Plasmodium* interactions.

Study system	Type	Purpose	Species			References
Experimental mosquito infections	*In vivo*	Transmission blocking assays Parasite life cycle Parasite and mosquito interactions Gene and protein functions related to the infection	Skin Feeding Assay	Any *Plasmodium* species. Mosquitoes feed on an infected host (mainly infected mice with *P.berghei* or *P.yoelii* laboratory strains)	*A.gambaie*, *A.stephensi*, *A.arabiensis*, among others, (laboratory or field-derived)	Blagborough et al., 2013; Bousema et al., 2013; Churcher et al., 2012; Miura et al., 2020
			Direct Membrane Feeding Assay	Any *Plasmodium* species. Gametocytes obtained from an infected host. Mosquitoes feed through a membrane device (*P. falciparum, P. vivax*, among others)		
			Standard Membrane Feeding Assay	Cultivable *Plasmodium* species able to produce gametocytes. Mosquitoes feed through a membrane device (mainly *P. falciparum* laboratory strains and field isolates)		
*In vitro* culture of Plasmodium mosquito stages	*In vitro*	Culture of *Plasmodium* stages in mosquito	*P. gallinaceum* *P. berghei* *P. yoelii* *P. vivax* *P. falicparum*			Warburg and Miller, 1992; Al-Olayan et al., 2002; Porter-Kelly et al., 2006; McClean et al., 2010; Eappen et al., 2022
Mosquito cell lines culture	*In vitro*	Haemocyte response to *Plasmodium* molecules	None			Akman-Anderson et al.; 2007; Pietri et al., 2015
Polyacrylamide gels	*In vitro*	Ookinete and sporozoite motility	*P. berghei*			Ripp et al., 2021
Baculovirus expression system in insect cells	*In vitro*	Mosquito-*Plasmodium* protein interactions	*P. falciparum – Anopheles gambiae*			Cui et al., 2020; Niu et al.,2021
Single cell RNA-seq in *Plasmodium*	*In vivo*	Profile the transcriptomics during plasmodium lifecycle in the mosquito	*P. berghei* *P. falciparum*			Howik et al., 2019; Witmer et al., 2021; Real et al., 2021; Mohammed et al., 2023
Single cell RNA-seq in mosquito	*In vivo*	Mosquito immune system in response to blood feeding or infection with *Plasmodium*. Mosquito midguts before and after a blood meal	*Anopheles gambiae – P berghei* *Anopheles gambiae* *Aedes aegypti*			Raddi et al., 2020; Kwon et al., 2021; Cui and Franz, 2020
Explanted midgut	*Ex vivo*	Ookinete locomotion and invasion through the midgut epithelium	*P. gallinaceum – Aedes aegypti* *P. berghei – A. gambiae and A. stephensi*			Zieler and Dvorak 2000; Vlachou et al., 2004

Apart from the methodology used to infect the mosquitoes, multiple parasite and mosquito traits can lead to variable rates of prevalence (percentage of infected mosquitoes) and intensity (number of parasites per mosquito) (Lefèvre et al., 2013; Vallejo et al., 2016; Simões et al., 2017). Parasite factors such as total gametocyte density, maturation stage, and sex ratio have been shown to impact mosquito infection (Churcher et al., 2013; Da et al., 2015; Bradley et al., 2018). There are also important differences between laboratory and field parasite isolates, and most laboratory reference strains have lost their ability to produce gametocytes (Omorou et al., 2022). Additionally, individual mosquito characteristics such as genotype, age, vector competence and susceptibility to infection, feeding and digestion behavior, and physiological state can result in a variable infection success which makes it difficult to reach a standardized and reproducible protocol (Miura et al., 2016). It has even been shown that the intensity of infection could be affected by the mosquito’s circadian clock (Habtewold et al., 2022). In the case of *Plasmodium falciparum* and its natural mosquito vectors, there are difficulties in establishing consistent, high-intensity infections in the laboratory. Various factors, like the vector competence of laboratory reared *vs*. field-caught mosquitoes, or the use of human *vs*. artificial serum, are critical parameters that may be difficult to overcome (Aguilar et al., 2005; Bousema et al., 2013). To reach high parasite numbers in SMFA and DMFA assays, a common approach is to use a non-natural interaction commonly between a rodent malaria parasite *Plasmodium berghei* and an *Anopheles* human-biting species. These combinations however are not representative of the natural mosquito environment to which a particular *Plasmodium* species is adapted, and therefore, the output of the interaction must be considered with caution (Boëte, 2005; Cohuet et al., 2006; Dong et al., 2006; Simões et al., 2017). Indeed, the intensity of infection, i.e. oocyst number per mosquito midgut, can differ between *P. falciparum* and *P. berghei* from tens to hundreds, respectively (Sinden et al. 2004; Aguilar et al., 2005). Mosquito immune responses to *P. falciparum* and *P. berghei* are also slightly different at the transcriptional level (Dong et al., 2006) and depending on the intensity of the infection different immune responses have been reported (Mendes et al., 2011; Simões et al., 2017). These differences can be explained by the fact that the non-natural combination is not constrained by the long-term co-evolution between naturally interacting species, where both organisms have adapted to each other, reducing virulence/resistance, in order to assure parasite survival while reducing mosquito fitness costs linked to the infection process (Shaw et al., 2022). Parameters that differ in natural and non-natural interactions, such as mosquito survival, fecundity and fertility, and parasite developmental rates and transmission efficiency have been reviewed elsewhere (Shaw et al. 2022).

Another challenge in the use of *in vivo* systems is the genetic manipulation of the parasite during the mosquito stages. Gene editing is essential to decipher gene and protein function, and widely used for parasite imaging and for drug/vaccine discovery assays (Okombo et al., 2021). To modify the genome of *Plasmodium*, transgenic parasite strains are better produced during the asexual blood stages because this is when parasites are replicative and haploid, which facilitates their selection and manipulation. Gene disruption strategies, have been essential to decipher parasite protein function playing an important role during mosquito infection. Examples include SOAP (Dessens et al., 2003) and CeLTOS (Kariu et al., 2006) proteins, both expressed in ookinete stages, and involved in parasite survival and dissemination; or transmission-blocking vaccine candidate antigens, like Pfs25, Pfs28, Pfs230 or Pfs48/45 among others (reviewed in Keleta et al., 2021). However, a major drawback for systematically assigning function is when the gene of interest (GOI) is essential for erythrocytic development or transmission, and therefore complete deletion (knockout) is not possible. At least half of the parasite genome has been described as essential for the completion of the asexual blood cycle for *P. berghei* (Bushell et al., 2017) and *P. falciparum* (Zhang et al., 2018). In order to modulate gene expression of essential genes, several conditional gene knockout and knockdown systems have been developed to study asexual blood-stage parasites. Detailed procedures of conditional expression systems, advantages and disadvantages, their applications at different parasite stages, and future perspectives have been recently reviewed (Kudyba et al., 2021; Briquet et al. 2022).

A few conditional approaches have been applied to mosquito stages. Promoter-swap strategies have been used in *P. berghei* gametocytes (Laurentino et al., 2011; Wall et al., 2018), ookinetes (Siden-Kiamos et al., 2011) and sporozoites (Ishino et al., 2019; Nozaki et al., 2020), which consists in a promoter-exchange of the GOI that maintains the expression in blood-stage parasites but become inactive in mosquito-stage parasites. This strategy, for instance, has allowed to study the role of rhoptry proteins in *P. berghei* sporozoites and salivary gland invasion, which are crucial for erythrocytes infection (Ishino et al., 2019; Nozaki et al., 2020). Another approach is the site-specific recombinase, Cre and Flippase, able to knockout the expression of a target DNA previously flanked with specific sequences LoxP and FRT, respectively. Depending on the orientation of these two-flanking locus, the recombinase enzyme will excise (same directions) or invert (opposite orientation) the targeted DNA. The activation of Flp/FRT recombinase system, which have been successfully implemented in *P. berghei*, is controlled through a stage-specific promoter, restricting the gene editing event to a particular parasite life-stage of interest (Carvalho et al., 2004; Combe et al., 2009; Lacroix et al., 2011). The DiCre system, which is expressed in two enzymatically inactive subunits, require the administration of rapamycin to induce heterodimerization and recombinase activation. Although this strategy makes the recombinase system more flexible, a correct delivery dose in the mosquito as well as the potential toxic effects of the compounds for the insect vector may be an important limitation. Recently, this system has been successfully used to delete essential genes in *P. berghei* prior to transmission to *A. stephensi* mosquitoes (Fernandes et al., 2022). The study demonstrates that silencing the Apical Membrane Antigen 1 (AMA1) and Rhoptry Neck Proteins (RONs) affects sporozoite invasion of salivary glands and invasion of mammalian hepatocytes. In other studies, although the conditional expression of the GOI was successfully achieved, a reduction in parasite number was observed, whether rapamycin was administrated to *A. stephensi* infected with *P. berghei* (Fernandes et al., 2020) or to blood stage *P. falciparum* parasites prior to infection (Tibúrcio et al., 2019). Indeed, it has been demonstrated that rapamycin, an inhibitor of the TOR pathway, boosts the mosquito *A. stephensi* immune response, hindering *P. berghei* development (Feng et al., 2021). Therefore, further investigation is needed to implement a conditional expression system in mosquitoes without affecting its physiology and allowing a tight control of gene expression at any stage of the developmental cycle.

Apart from the pros and cons of different strategies used to study gene function in *Plasmodium* parasites, *in vivo* RNA interference (RNAi) and CRISPR/Cas9 gene silencing approaches in mosquitoes have proven to be very useful in advancing knowledge of the function of mosquito proteins and their potential interactions with malaria parasites. The main strategy used to knock down the expression of a given mosquito gene is using RNAi (Catteruccia and Levashina, 2009). This can be achieved by injecting gene-specific double-stranded RNA (dsRNA) into the adult mosquito or by expressing dsRNA *in situ* from transgenes integrated into the mosquito genome. The exogenous RNA then binds to the homologous mRNA of the candidate gene and causes its degradation. The impact of mosquito gene silencing on parasite survival using the RNAi strategy has uncovered the important function of many proteins involved in parasite midgut invasion, such as AnAPN1 (Dinglasan et al., 2007), FREP1 (Zhang et al., 2015), and P47Rec (Molina-Cruz et al., 2020), and also genes related to mosquito immunity, like LRIM (Osta et al 2004; Billingsley et al., 2021), TEP1 (Blandin et al., 2004), FBN9 and FBN30 (Dong and Dimopoulos, 2009; Li et al., 2013). The RNA delivery injection method is more widely used because it allows gene function to be assessed in a relatively short time, but it requires large numbers of mosquitoes and their physical manipulation can cause damage and stress (Taracena et al., 2022). Furthermore, this type of gene silencing is transient and time-limited. The generation of transgenic lines expressing RNAi, on the other hand, provides stable expression, a supply of mutant mosquitoes and a major control of knockdown using tissue-specific promoters, however it is labor intensive and requires longer periods of time (Catteruccia and Levashina, 2009). In both cases efficiency depends on the endogenous levels of the transcripts and whether expression is restricted to the target tissue or is more widespread. CRISPR/Cas9 gene editing allows a complete gene silencing at the DNA level and has been used to knock out genes in mosquitoes like *A. gambiae* FREP1 (Dong et al., 2018) and *A. stephensi* LRIM (Inbar et al., 2021), however, in both studies this strategy resulted in fitness costs, affecting mosquito’s development, fecundity and survival.

## 3 *In vitro* systems

*In vitro* culture systems represent a simplification of the biological complexity of an organism but are very useful and necessary to study how this complexity is generated and organized and how it functions. That is, they provide a controlled and isolated environment that permits more detailed analysis and easier manipulation. In the context of human infectious diseases research, *in vitro* systems allow to study the infection process and host-parasite interactions avoiding human experimentation.

In the malaria field, *in vitro* culture is the gold standard for the study of the parasite intraerythrocytic cycle in humans that has led to the identification of host and parasite factors that contribute to infection (Venugopal et al., 2020). This system has also been widely used for the high throughput screening of novel chemotherapeutics. The *in vitro* culture of the mosquito stages on the other hand, has been far more complicated and this area is still under development.

The major hurdle for *in vitro* culture of mosquito-stages, is that parasite development in the mosquito does not occur intracellularly and therefore the variety of environments, tissues and cellular types involved in the interactions are much more difficult to reproduce *in vitro*.

Although in reality there is no *in vitro* system that recapitulates faithfully the mosquito environment, the temporary culture of the parasite outside the vector is now possible for many *Plasmodium* species (**Table 1**). Important developments have been achieved in the two rodent malaria parasites: *P. berghei* (Al-Olayan et al., 2002) and *P. yoelii* (Porter-Kelley et al., 2006), while the human malaria parasite *P. falciparum* remains more challenging, at least until recently (Eappen et al., 2022). Advancements in the culture of mosquito stages have opened the door to functional and structural investigations of the sporogonic cycle (Zeeshan et al., 2021) as well as drug screening against mosquito stages (Azevedo et al., 2017), and represent the first step for the development of more complex *in vitro* systems. In the following sub-sections, the most important advances in this area are presented.

### 3.1 *In vitro* development of *Plasmodium* mosquito stages

The *in vitro* development of *Plasmodium* mosquito stages has been in the spotlight of research for a long time. The entire sporogonic cycle, from gametocytes to sporozoite, achieved *in vitro* was first reported in 1992 for *P. gallinaceum* (Warburg and Miller, 1992) followed by other *Plasmodium* species of both human(Warburg and Schneider, 1993) and non-human (Al-Olayan et al., 2002; Porter-Kelley et al., 2006). The full sporogonic development of *P. falciparum* was first described in 1993 (Warburg and Schneider, 1993), but the method was not reproducible, and the low recovery of parasites after each transformation step, has limited its application. Since then, there have been several attempts to improve the system. Different conditions have been tested and upgraded with more or less success and efficiency such as the culture medium composition, co-cultivation with insect cells and the presence of Matrigel substrate or other components simulating the basal lamina (Ghosh et al., 2010; Itsara et al., 2018; Siciliano et al., 2020).

Some conclusions can be drawn from these studies. The sporogonic development *in vitro* is achieved in two differentiated steps: (1) the gametes activation until ookinete development, followed by (2) oocyst differentiation and sporozoite production (**Figure 2C**). For the *in vitro* exflagellation and ookinete development, despite some variations, stage V gametocytes are cultured with RPMI medium (supplemented with fetal bovine serum, sodium bicarbonate and hypoxanthine among others) together with factors or conditions that are known to trigger gametocyte differentiation such as the presence of xanthurenic acid or a temperature drop (Billker et al., 1998; Garcia et al., 1998). This method has been widely used in *P. berghei* and has allowed to study many biological processes during sexual development like ookinete formation and invasion, which has been translated to a better understanding of transmission biology of this parasite compared to other species (Guttery et al., 2022). On the other hand, *in vitro* conversion of ookinete to oocyst and sporozoite production are more challenging as the *in vivo* setting in which these processes take place is more complex and the triggering factors as well as the regulatory mechanisms of cell division and differentiation remain mostly unknown (**Figure 1**). Oocyst differentiation begins when the ookinete reaches the basal lamina, but oocyst maturation until sporozoite release requires a long period of time (around 20 days) and involves multiple mitotic divisions and interactions with the surrounding midgut epithelial cells. To mimic these steps *in vitro*, once ookinetes are obtained, they are recovered and cultured in different conditions. Generally, a supplemented Schneider’s medium is used with the presence of insect cells, such *Drosophila melanogaster* S2 cells, and Matrigel or similar substrates. It has been shown that the use of collagen-based matrices and feeder cells improve the conversion rates and the sporozoite production (Al-Olayan et al., 2002; Porter-Kelley et al., 2006; Azevedo et al., 2017). Probably, the role of collagen-based matrices, similar to the midgut basement membrane, is to allow ookinete attachment and enhance oocyst differentiation. More unclear is the role played by insect cells, which might be related to factors secreted that may act as trigger factors for oocyst developmental progression. Despite these advances, mainly in *P. berghei* and *P. yoelii*, oocyst maintenance and differentiation *in vitro* remain challenging, parasite recovery rate is very low and it decreases further over time.

Recently, an improved approach for the complete *P. falciparum* sporogonic development has been described (Eappen et al., 2022). The authors increased considerably the yield of sporozoites obtained, which in addition, were able to infect and transit to blood stages. In that study, sporogonic development was achieved in three steps with specific conditions: exflagellation, ookinete development, and oocyst transformation. Thanks to the presence of S2 feeder cells and Matrigel a high transformation rate from gametocyte to oocyst was obtained. Although the conversion efficiency from oocyst to sporozoite was lower *in vitro* than in the mosquito, the final conversion rate (from gametocytes to sporozoites) was 7.4-fold higher *in vitro*. However, as a caveat, due to the lower conversion rate of oocyst to sporozoites *in vitro*, sporozoite release was forced by mechanical dissociation of mature oocyst, suggesting that still unknown factors are required for a normal development. Furthermore, the sporozoites obtained *in vitro* showed attenuation of their infectivity at the late liver stage, probably indicating that as observed *in vivo*, sporozoite infectivity may be slightly compromised if they do not pass through the salivary glands (Touray et al., 1992; Sato et al. 2014). Although overall gene expression by RNA-seq was similar between sporozoites produced *in vitro* and *in vivo*, many reads were not parasite-specific due to the presence of S2 cells. Altogether, further investigation is needed to decipher observed differences in infectivity.

Another recent development in this area is the use of *in vitro* platforms to study the motility of ookinetes and sporozoites, which is essential for malaria transmission (Ramírez-Flores et al., 2022). The use of polyacrylamide gels that can be adjusted in elasticity and pore size, allows a more accurate simulation of different mosquito tissues and microenvironments. Accordingly, it has been observed that both ookinete and sporozoite motility and migration paths show differences depending on substrate characteristics (Ripp et al., 2021; Vaughan, 2021).

Altogether, for a successful *in vitro* culture of *Plasmodium* mosquito stages, a major improvement would be the development of new two-dimensional (2D) or three-dimensional (3D) culture systems that enable the sporogonic cycle in a continuous manner and that recapitulate the structural and physiological conditions of the mosquito environment more faithfully.

### 3.2 Other *in vitro* systems in malaria

Although several mosquito cell lines exist (Walker et al., 2014) none of them is suitable to study the *Plasmodium*-mosquito interactions. Several of the cell lines that have been developed have hemocyte-like properties and have been used to study mosquito immunity (Mishra et al., 2022). Some studies have used *Plasmodium*-derived molecules to study the mosquito cells’ immune response, but none has co-cultured the mosquito cell lines with the parasite to study their interactions (Akman-Anderson et al. 2007; Pietri et al., 2015).

Another *in vitro* strategy that has recently been used to uncover *P. falciparum* and *A. gambiae* protein interactions is the baculovirus expression system in insect cells. This system allows the production of recombinant proteins and has been used to discover both parasite (Niu et al., 2021) and mosquito proteins (Cui et al., 2020) involved in mosquito infection. For such purpose, they chose proteins that may directly interact in the midgut lumen: proteins with signal peptides, whose genes are up-regulated after the blood meal in mosquitoes, or are abundantly expressed at sexual stages in parasites. Once the candidate genes were cloned and expressed in the baculovirus system, the recombinant proteins were used in an ELISA assay with mosquito midgut lysates or specific *P. falciparum* stages to detect potential protein interactions. The effects of the protein interactions during the infection process need then to be confirmed *in vivo*. By knocking down the expression of mosquito candidate proteins using RNAi, and analyzing the oocyst number developed after *in vivo* infection, it was possible to uncover both mosquito proteins that protect against infection and proteins that facilitate parasite invasion (Cui et al., 2020). On the other hand, the function of a parasite protein candidate, Pfs16, was assessed using an antibody anti-Pfs16 which significantly reduced the number of oocysts (Niu et al., 2021). Altogether, this strategy allows the detection of potential targets to block malaria transmission.

## 4 Single cell technologies

Infection is a dynamic process in which parasite and mosquito gene expression patterns and their regulation change spatially and temporally, allowing the parasite to transit between life-stages and adapt to within-host conditions, and the mosquito to respond to an infection by a particular parasite genotype/phenotype. These interactions and their consequences are best represented in an *in vivo* system, but profiling “in bulk” the genome, the epigenome or the transcriptome of the parasite or the mosquito using infected tissues, where many cell types are present, may distort and bias the results. Recent advances in single cell technologies have led to a breakthrough in the analysis of heterogenous samples and environments, allowing a deep understanding of host-parasite interactions at the single cell level (Afriat et al., 2022). With this approach the genetic diversity of an infection can be captured, mapping out gene expression throughout the developmental timeline, detecting key expression and regulatory processes and predicting gene function by association with other co-expressed and functionally annotated genes (Real et al., 2021).

Single cell RNA-seq approaches have been applied widely in different *Plasmodium* species and developmental stages (reviewed in Real and Mancio-Silva, 2022). The data obtained from some of these studies are part of the Malaria Cell Atlas project, which aims to build up a reference map of the parasite transcriptome during its entire development (Howick et al., 2019; Nötzel and Kafsack, 2021).Single-cell transcriptomic data is available for *P. berghei* (Howick et al., 2019; Witmer et al., 2021) and *P. falciparum* (Real et al., 2021; Mohammed et al., 2023) during the life cycle inside the mosquito.

However, regarding mosquitoes, single cell approaches have only been used to study the mosquito immune system in *A. gambiae* and *Aedes aegypti* (Raddi et al., 2020; Kwon et al., 2021). In *A. aegypti* another pioneer study applied single cell RNA-seq to mosquito midguts, before and after a blood meal, revealing changes in cellular composition and transcriptional profile due to infection (Cui and Franz, 2020). This demonstrates that if applied to midguts and salivary glands of *Anopheles* before and after infection, this technology could shed new light on mosquito responses at the single cell level, and reveal the changes induced by the parasite in the expression of different mosquito cell types, as well as the strategies and mechanisms used by the parasite to migrate through the mosquito’s body.

A promising approach offered by single cell approaches is to study parasite and mosquito transcriptomic and epigenomics changes simultaneously. The dual scRNA-seq strategy of infected cells has been used to study parasite-host interactions during the erythrocytic cycle and in the liver (Hentzschel et al., 2022; Mancio-Silva et al., 2022). However, the spatial context is lost with the single cell approaches and, in order to study interactions, additional techniques are required to link tissue distribution and transcriptional profiles. Different spatial transcriptomics strategies exist nowadays providing a coordinate map of the distribution of cells in a tissue based on specific gene sets (Williams et al., 2022). If this data is integrated with scRNA-seq it is possible to associate transcriptomic information with specific spatial localization in the native tissue (Longo et al., 2021). In a recent study, scRNA-seq and single-molecule fluorescence *in situ* hybridization (smFISH) data have been combined to study *P. berghei* development in the mouse liver (Afriat et al., 2022). A spatial profile of the interactions between parasite and host cells was achieved, identifying differences in parasite growth and survival in distinct zones. Nevertheless, the application of this technology in the field is still in its infancy.

## 5 *Ex vivo* culture systems

*Ex vivo* systems aim to represent the cellular complexity of organs or tissues found *in vivo* using *in vitro* conditions (outside the organism). These 3D culture systems allow standardized and controlled experimentation, while providing a closer representation of the *in vivo* situation.

One could divide these systems into: tissue explants and engineered tissues and organs (organoids). Tissue explant refers to the culture of small pieces of a tissue extracted from an animal or organ. Organoids are tiny, self-organized three-dimensional tissue cultures that are derived from stem cells. Such cultures can be crafted to replicate much of the complexity of an organ, or to exhibit selected aspects of it.

Mammalian organoids and explant tissues have been widely used to study interactions of many infectious diseases, including apicomplexan parasites like *Plasmodium*, *Toxoplasma*, *Cryptosporidium* and *Eimeria* (Dutta and Clevers, 2017; Ramírez-Flores et al., 2022). These systems allow us to study pathogen biology and host interactions in a more accessible way, overcoming other limitations of the systems mentioned above. It opens up the possibility of performing live-imaging experiments, facilitates gene editing strategies and even allows the culture of organisms that are difficult to grow *in vitro* in traditional 2D culture systems, which lack cellular architecture, extracellular microenvironment and poorly represent the natural niche. Unfortunately, these advances have not been translated to insect models. This is in spite of providing new opportunities to study unknown aspects of parasite-vector interactions but also discover and test new molecules that block pathogen transmission.

### 5.1 Explanted tissues

In the malaria field, explanted midgut tissues (Zieler and Dvorak, 2000) and entire intact midguts (Vlachou et al., 2004) have been used to study ookinete locomotion and invasion through the epithelium (**Table 1**). A culture system was developed to maintain the tissue alive while observing the invasion process of ookinetes by microscopy. The tissue viability and cell apoptosis were assessed with dyes and morphological observation, estimating a lifespan of 2-3 hours. While this strategy might be useful for short time processes, i.e. gametocyte activation or zygote formation, mantaining mosquito tissues alive over a longer period of time still represents a hurdle. This limitation does not affect many mammalian tissues, in which the *ex vivo* culture strategy has been widely used to study diverse physiological and pathological processes during longer periods of time (Randall et al. 2011; Russo et al., 2016). For example, it has been applied to study infection by the apicomplexan parasite *Cryptosporidium parvum*. In this case, the murine intestine explant remained alive in culture for 35 days (Baydoun et al., 2017). This reflects a much better understanding of the conditions required for *in vitro* culture of mammalian cells and tissues compared to insects.

An approach that permits to assess the viability of an explanted tissue or organ would be very valuable in the context of malaria. Antonello et al. developed a method to analyse the dynamics of the intestinal epithelium in *Drosophila*, named ReDDM system (Repressible Dual Differential-stability Markers) (Antonello et al., 2015). This method uses the Gal4/UAS system to control the expression of two different fluorescently labelled proteins, with short and long half-lives. When this system is controlled by the expression of a gene only active in progenitor cells, due to the different half-lives of the tagged proteins, it is possible to track cell turnover and distinguish the newly differentiated cells. Interestingly, an improved explant culture of Drosophila midgut has been recently reported (**Figure 2D**) (Marchetti et al. 2022). The *ex vivo* system sustains the organ alive for up to 3 days and allows live-imaging during that time, enabling monitoring of the tissue epithelial dynamics. We envision that, if leveraged to mosquitoes, these approaches might be a promising tool to monitor the midgut viability and homeostatic activity during an infection process.

### 5.2 Stem cell technologies and organoids

The advent of stem cells has opened up exciting new applications and opportunities to understand disease mechanisms, recapitulate cellular systems and genetic characteristics (Pance, 2021). Stem cell research boomed in mammalian studies with the capacity to generate induced pluripotent stem (iPS) cell lines and differentiate into specific cell types, making it possible to generate traditional *in vitro* cell culture of a single cell type or more complex multicellular structures that recapitulate the characteristics of an organ, also called organoids (**Figure 2E**). Another important contribution of these systems is the storage of cell lines, facilitating experimental procedures and also providing greater homogeneity and tractability to the studies performed (Hanna and Hubel, 2009). Such advances have been scarce in insects, including main disease vectors.

As a first step, however, the identification of stem cell types in a variety of insects has been reported (Corley and Lavine, 2006). In the case of mosquitoes, midgut stem cells from the house mosquito *Culex pipiens* have been isolated and cultured, though for a limited period of time (Wassim et al., 2014). This pioneer work demonstrates that it is possible to obtain, culture and store insect stem cells, raising exciting possibilities for the generation of longer-term stem cells cultures capable of supporting a pathogen infection. Nevertheless, similar stem cell types from malaria mosquito vectors are still lacking.

Recently stem cells technologies have been applied to derive different types of human red blood cells to culture the parasites, erythroid precursors and genetically modified mature erythrocytes. This approach has enabled a better understanding of parasite invasion and pathogenesis and offered the possibility of studying patient-derived cell lines that can be preserved and manipulated to understand the impact of genetic variation on the disease (Pance et al., 2021). In the mosquito, however, one great hurdle of this novel technology is that the mosquito stages of *Plasmodium* parasites are extracellular. In this case, stem cell-derived organoids would be much better suited to create an easy manipulable environment simulating the complexity and variety of tissues. Mammalian gut organoids have been recently engineered aiming to harbor and culture unicellular as well as multicellular pathogens (Pance, 2021; Ramírez-Flores et al., 2022) and an application to insects has been suggested (Swevers et al., 2021).

Organoids can be generated from iPS cells, embryonic stem cells or adult stem cells from specific tissues (**Figure 2E**). The formation of organoids is based on the culture of stem cells with an extracellular matrix, which allows tridimensional structure formation, and niche factors that stimulate self-renewal and induce cellular differentiation. Multiple mammalian organoids such intestines (Almeqdadi et al., 2019; Nikolaev et al., 2020) and salivary glands (Pringle et al., 2016; Tanaka et al., 2018) have been created among others.

The insect midgut epithelium is of particular interest because of its role in nutrition, digestion and immunity as well as a niche for microbiota and an interphase of parasite interactions. The dynamics of the gut epithelium and identification of some of its constituting cell types have been described in *Drosophila* as a model organism, including progenitor cells (Bonfini et al. 2016). Although the *Drosophila* midgut epithelium shares similarities with human intestine, such as cell types and functions, as well as molecular signaling pathways which drive intestinal stem cell (ISC) proliferation and differentiation (Kaur et al., 2018; Capo et al. 2019), the use of stem cell technologies in flies has not been reported to date. Compared to *Drosophila*, the midgut epithelial dynamics of mosquitoes have received much less attention. However, some studies have started filling this gap, highlighting the potential of this system (Hixson et al. 2021).

The mosquito midgut comprises four main cell types: differentiated enterocytes (ECs) and enteroendocrine cells (EEs), and undifferentiated progenitor cells (ISC and enteroblasts, EBs). The common features in the midgut cell composition and cell type markers between *Aedes aegypti* and *Drosophila* was recently reported using scRNA-seq (Cui and Franz, 2020). It was also demonstrated that the mosquito midgut epithelium is a dynamic tissue which changes its cell composition after a blood meal, indicating a proliferative and differentiation response. Furthermore, proliferative cells from the mosquito gut that are responsive to damage and able to repair the epithelium have also been studied (Janeh et al. 2017), although not all mosquito species respond to damage in the same way (Janeh et al. 2019). The signals involved in the regulation of the ISC are poorly understood. It has been speculated that the hormone 20 hydroxyecdysone (20E), which increases after a blood meal, could stimulate the ISC proliferation as has been described in *Drosophila* (Hixson et al. 2021). Similarly, induced pathways after mosquito gut damage such as Jak/Stat, EGFR and Delta-Notch signaling (Janeh et al. 2017; Taracena et al., 2018) may be involved in the epithelium regeneration response. However, much research is required to decipher their role in midgut homeostasis, as well as identify the ISC niche factors that triggers proliferation and differentiation into a specific cell type. Developing these systems and understanding these processes will represent an invaluable contribution to the study of vector-parasite interactions and unravelling of malaria transmission.

## 6 Concluding remarks

A major complication of any *in vitro* system to study *Plasmodium*-mosquito interactions is that the parasite in the mosquito is extracellular. Indeed, nowadays there is no single, well-established protocol for the complete *in vitro* sporogonic development of any *Plasmodium* species. This is probably because current systems fail to reproduce faithfully all the different environments in the mosquito and also because several key triggering factors enabling the progression of the parasite life cycle in the mosquito still need to be unraveled.

The development of *ex vivo* systems that recapitulate the complexity of the mosquito environment and can be easily handled *in vitro*, may help to overcome some of the limitations of the current *in vitro* and *in vivo* systems, facilitating genome editing of the parasite and assuring high performance and reproducibility of the experiments. Explanted tissues represent a powerful alternative; however, its use is still limited by the amount of time the organ preserves its functionality and integrity, which in the case of mosquitoes is currently unknown.

The availability of proliferative and stem cells from mosquitoes will enable the design of novel 2D and 3D culture systems, such as organoids, simulating mosquito midgut and salivary glands, to support development and transmission of the vector stages of many human parasites, including *Plasmodium*. Renewed efforts in transdisciplinary research and stem cell technologies are needed to identify, isolate, culture and differentiate these cells, and develop three-dimensional structures to facilitate the study of interactions between parasites such as *Plasmodium* and their vectors. Such developments will be fundamental in the quest for novel tools to control infectious diseases.

## Author contributions

Conceptualization: MP-M and EG-D. Original draft preparation: MP-M, EG-D, and AP revised it critically and added important intellectual content. All authors contributed to the article and approved the submitted version.
